# Microbial diversity and proxy species for human impact in Italian karst caves

**DOI:** 10.1038/s41598-022-26511-5

**Published:** 2023-01-13

**Authors:** Federico Biagioli, Claudia Coleine, Elena Piano, Giuseppe Nicolosi, Anna Poli, Valeria Prigione, Andrea Zanellati, Cristina Varese, Marco Isaia, Laura Selbmann

**Affiliations:** 1 Department of Ecological and Biological Sciences, University of Tuscia, 01100 Viterbo, Italy; 2 Department of Life Sciences and System Biology, University of Torino, 10123 Turin, Italy; 3 Department of Life Sciences and System Biology, Mycotheca Universitatis Taurinensis, University of Torino, 10125 Turin, Italy; 4 Mycological Section, Italian National Antarctic Museum (MNA), 16121 Genoa, Italy

**Keywords:** Ecology, Microbiology

## Abstract

To date, the highly adapted cave microbial communities are challenged by the expanding anthropization of these subterranean habitats. Although recent advances in characterizing show-caves microbiome composition and functionality, the anthropic effect on promoting the establishment, or reducing the presence of specific microbial guilds has never been studied in detail. This work aims to investigate the whole microbiome (Fungi, Algae, Bacteria and Archaea) of four Italian show-caves, displaying different environmental and geo-morphological conditions and one recently discovered natural cave to highlight potential human-induced microbial traits alterations. Results indicate how show-caves share common microbial traits in contrast to the natural one; the first are characterized by microorganisms related to outdoor environment and/or capable of exploiting extra inputs of organic matter eventually supplied by tourist flows (i.e. *Chaetomium* and *Phoma* for fungi and *Pseudomonas* for bacteria). Yet, variation in microalgae assemblage composition was reported in show-caves, probably related to the effect of the artificial lighting. This study provides insights into the potential microbiome cave contamination by human-related bacteria (e.g. *Lactobacillus* and *Staphylococcus*) and commensal/opportunistic human associated fungi (e.g. *Candida*) and dermatophytes. This work is critical to untangle caves microbiome towards management and conservation of these fragile ecosystems.

## Introduction

Caves are spatially confined, subsurface environments widespread on Earth, where peculiar abiotic factors (i.e. oligotrophy, total darkness and high mineral concentrations) and microclimatic conditions (i.e. scarcely fluctuating low temperature and very high humidity)^1,2^ impose special adaptations to microbial life-forms^3^. Microbial assemblages in caves include archaea, bacteria, fungi and other micro-eukaryotes^4^; these highly adapted microbial communities represent the living-backbone of cave ecosystems and play a key role in shaping structures (via direct and indirect metabolic activities) and sustaining trophic networks^5–7^. They live in constant balance between coping with unfavorable environmental conditions and maintaining microbe-microbe interactions, either competitive or cooperative^1,8^.

Yet, the growing speleological and touristic interest in these underground settings has led to a major anthropization of caves (thereafter show-caves), which have been turned into real attractions. To date, show-caves, with 1440 sites in 148 countries, among which 60 are in Italy (www.showcaves.com), represent an expanding worldwide scenario that results in a constant anthropization of a large number of natural hypogean settings. The presence of logistic interventions, such as the establishment of walkways, barriers and artificial lighting systems, coupled with tourist flows may alter the pristine local environmental conditions, by changing the local microclimate and introducing nutrients (i.e. soil, hair, skin, lint from clothing) and allochthonous microbial species^9–13^.

Recently, researchers started to characterize show-caves microbiome, analyzing different types of samples (sediments, speleothems, and biofilms) and comparing natural and show-caves to discern a potential human impact on microbial composition and functionality [e.g.^13–17^]. Potential bioindicators of altered conditions due to human activities have been identified in showcaves; for instance, the proliferation of lampenflora component, due to artificial light, is more abundant but generally less diverse than in natural caves^18^. Although some predominant microbial taxa reported in pristine settings have been detected in anthropized caves^8,13^, the effect of human impact on promoting the establishment or reducing the presence of specific microbial traits has never been investigated in detail. Therefore, the consequence of tourist flows and human activities in general on microbial assemblages composition remains largely unknown.

To address this knowledge gap, our study intends to provide novel comprehensive insights into the microbial diversity and composition of 5 caves that include 4 show-caves and 1 recently discovered natural cave, displaying different environmental and geo-morphological conditions across the Italian peninsula. Through taxonomic comparison, we have characterized the principal microbial traits found in anthropized and pristine habitats, starting to shed light on potential microbial taxa that may be considered as indicative of human impact.

## Results

### Microbial community composition in cave sediments

The ITS1 dataset generated 5,793,980 raw sequence reads, resulting in 5,458,895 gene quality-filtered reads, ranging from 1252 up to 540,803 per sample. After singletons and rare taxa (< 5 reads) removal (1108 out of 10,595 ASVs total), a total of 9176 high-quality ASVs were obtained (Table S1). A total of 5,453,881 raw reads were generated from 16S rDNA dataset and accounted for a total of 4,806,902, which were grouped into 31,878 ASVs (out of a total of 65,037 ASVs) after quality filtering, with sequencing depths between samples ranging from 2066 to 265,442 reads .

Subsequently, the total 16S dataset was splitted by grouping the bacterial (31,015 ASVs) and archaeal (863 ASVs) ASVs separately for the downstream analyses (Table S2; Table S3). The 18S dataset was processed through a specific evaluation, extracting and analyzing only the sequences related to the microalgal component. A total of 97 ASVs were mapped for a total of 10,084 quality filtered reads, spread over 4 different groups (Alveolata, Chloroplastida, Chromista and Stramenopiles), using the Class taxonomic rank as the threshold for taxonomic identification as a consequence of the weak accuracy in the assignment towards higher ranks (Table S4).

**Figure 1:**
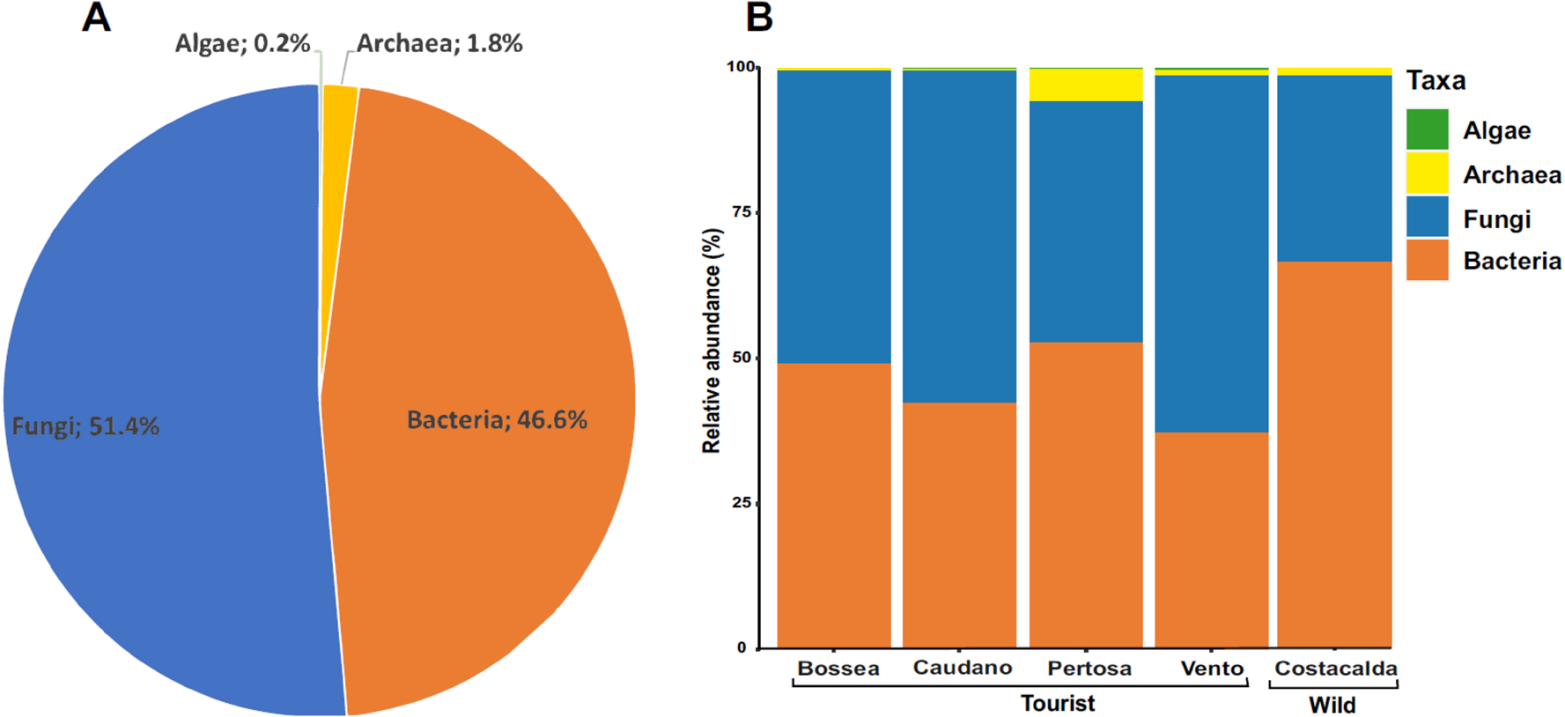
Total microbial cave community composition: as a unique dataset (**A**) and per single cave perspective (**B**).

Considering the total composition of microbial communities in cave sediments as a unique dataset (Fig. 1A), fungi represented the most abundant group (51.4%), followed by bacteria (46.6%), while archaea (1.8%) and micro-algae (0.2%) constituted minor fractions.

When analyzing the assemblage composition of each cave individually (Fig. 1B), Bossea cave was characterized by an almost equal presence of fungi (50.22%) and bacteria (49.16%). Caudano and Vento caves were dominated by fungi (57.17% and 61.46%, respectively), while the bacterial community accounted for 42.37 and 37.22% of the total assemblage, respectively. Conversely, Costacalda and the Pertosa-Auletta caves showed a higher bacterial presence (66.66% and 52.70%, respectively) compared to fungi (32.05 and 41.48%, respectively). Concerning the archaea domain, Pertosa-Auletta cave was the most representative with 5.74% of the total microbial assemblage, followed by Costacalda (1.27%), Vento (0.85%), Bossea (0.52%) and Caudano (0.41%) caves. The algal component was scarcely represented along all caves, with a relative abundance ranging from 0.47 for Vento cave to 0.01% for Costacalda cave.

### Fungal community composition

**Figure 2:**
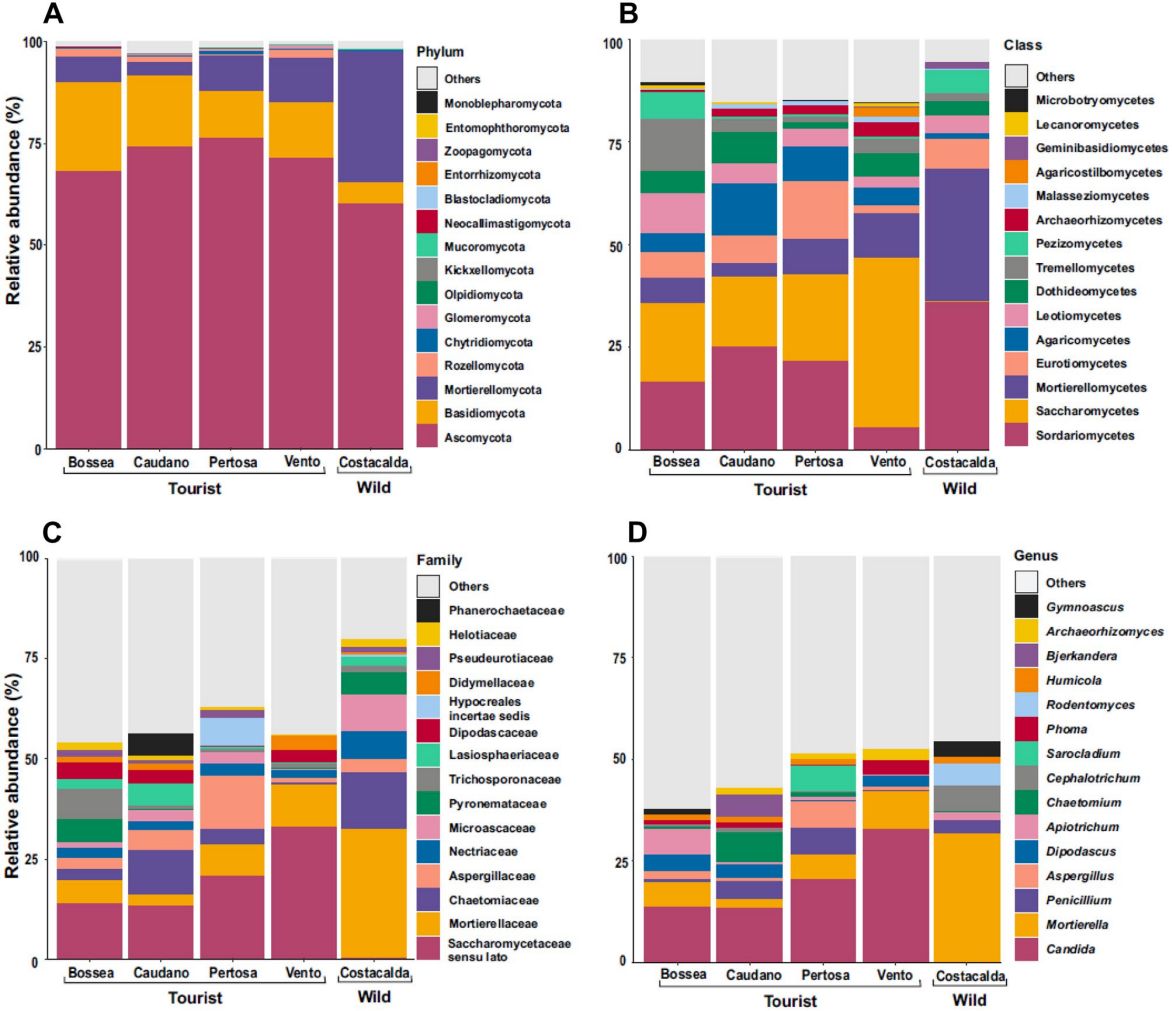
Fungal community composition of 5 examined caves: Bossea, Caudano Pertosa-Auletta, Vento (show-caves) and Costacalda (wild cave). The top 15 most abundant taxa are shown for taxonomic ranks: phylum (**A**), class (**B**), family (**C**) and genus (**D**).

In all caves*,* the dominant phylum was Ascomycota (Fig. 2A) (ranging from 60% in Costacalda to 76.25% in Pertosa Auletta), followed by Basidiomycota and Mortierellomycota. A broad different distribution of fungal taxa pattern emerged between the four show -caves and the wild cave (Fig. 2A,B,C,D). Notably, the genus *Mortierella* (Fig. 2D) was particularly abundant in the Costacalda wild cave (up to 31.8%), while in the show- caves did not exceed 10% (from 2.31% in Caudano up to 9.30% in Vento). The opposite was observed for the genus *Candida* (class Saccharomycetes), which was more abundant in the tourist caves (ranging from 13.36% in Caudano to 37.32% in Vento). Also, the genus *Dipodascus* was mainly present in the anthropized settings, even if at much lower extent compared to *Candida*, ranging from 0.14% in Pertosa-Auletta to 4% in Bossea. The genus *Archaeorhizomyces* (class Archaeorhizomycetes) was reported in show- caves only, albeit with scarce relative abundance (e.g. 0.01% Bossea up to 2.70% Vento). A number of cave-specific genera in the class Sordariomycetes have been recorded: for instance, *Chaetomium* (7.50%) in Caudano, *Sarocladium* (6.4%) in Pertosa-Auletta, *Cephalotrichum* (6.30%) and *Rodentomyces* (5.60%) in Costacalda ; differently, the genus *Humicola* (average abundance 1.39%) was found in all caves (Fig. 2). The class Eurotiomycetes was mainly represented by the genera *Aspergillus* (ranging from 0.01% in Costacalda to 6.77% in Pertosa-Auletta) and *Penicillium* (ranging from 0.17% in Vento to 6.45% in Pertosa-Auletta), while the genus *Gymnoascus* was most abundant in Costacalda and Bossea caves, accounting for 3.72% and 1.50% respectively. Likewise, the genera *Tetracladium* and *Pseudogymnoascus* (not shown in the Top 15) were the dominant members of Leotiomycetes throughout the dataset, up to1.96% and 1.12% in Costacalda , respectively (Fig. 2D). Agaricomycetes class was particularly abundant in show-caves (Caudano 12.6%, Pertosa-Auletta 8.25%, Bossea 4.44%, Vento 4.30%, Costacalda 1.2%), with the genus *Bjerkandera* being the only one reported among the top 15 (Fig. 2D). Yet, despite a considerable proportion of classes Tremellomycetes (ranging from 1.41% at Pertosa-Auletta to 12.7% at Bossea) and Dothideomycetes (ranging from 1.64% at Pertosa-Auletta to 7.50% at Caudano) among caves, they were mainly represented by two genera, i.e. *Apiotrichum* (Trichosporonaceae) and *Phoma* (Didymellaceae). *Apiotrichum* was spotted across all sites investigated, while *Phoma* appeared more prevalent among tourist caves (Vento: 3.45%, Caudano: 1.51%, Bossea: 1.10%, Pertosa-Auletta: 0.07%, Costacalda: 0.004%). Some other fungal classes showed different abundance patterns between tourist and wild cave, such as: Malasseziomycetes, (over 1% in show-caves vs. 0.15% of Costacalda), Microbotryomycetes (Bossea: 0.93%, Vento: 0.3%, Caudano: 0.16%, Pertosa-Auletta: 0.13%, vs. Costacalda: < 0.001%) and Geminibasidiomycetes (Costacalda: 1.52%, vs. all tourist caves: < 0.2%). Finally, lichenized fungi class of Lecanoromycetes was especially reported in Bossea (0.85%) and Vento (0.52%) caves.

### Bacterial community composition

**Figure 3:**
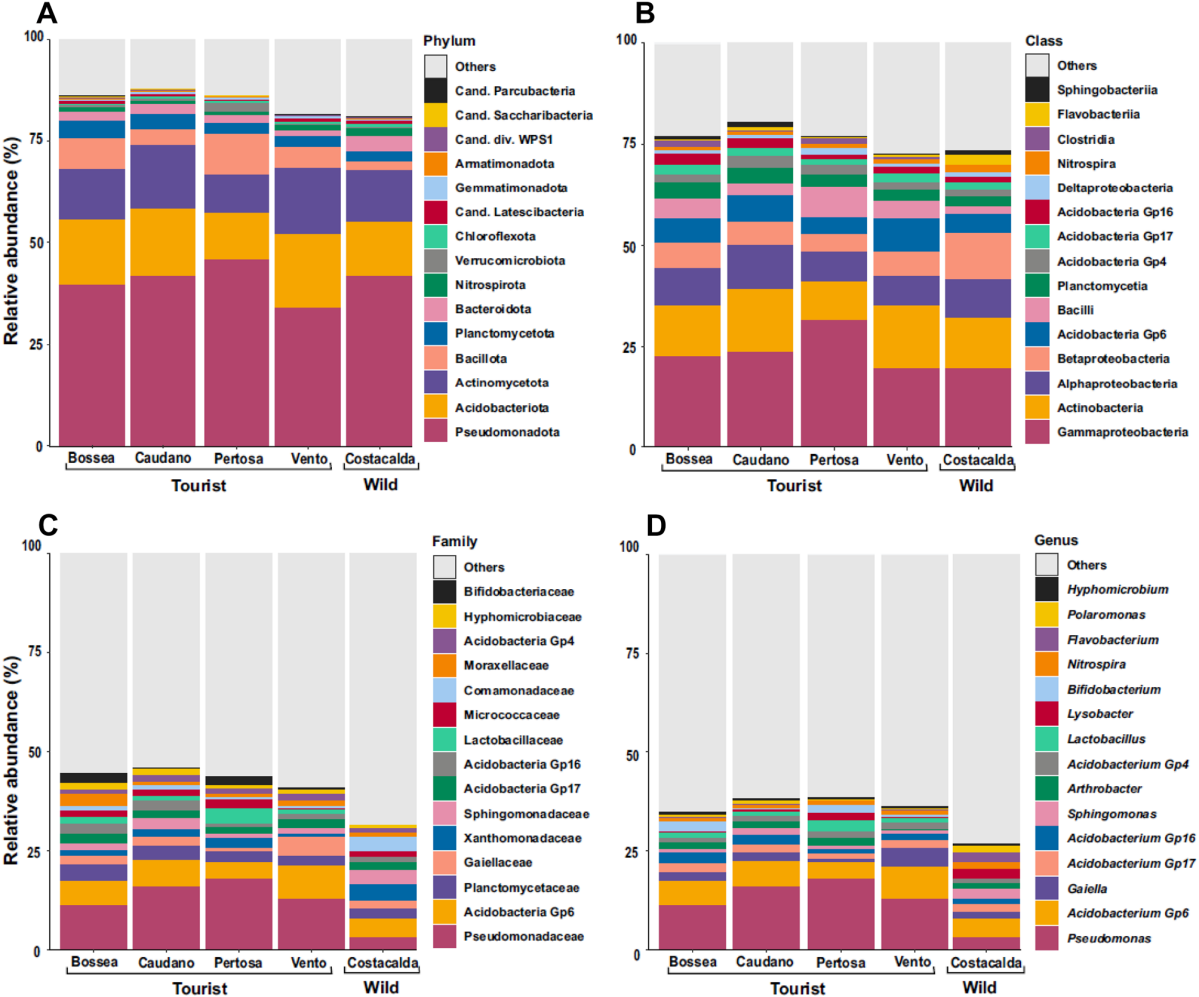
Bacterial community composition of 5 caves studied: Bossea, Caudano Pertosa-Auletta, Vento (show-caves) and Costacalda (wild cave). The top 15 most abundant taxa are shown for taxonomic ranks: phylum (**A**), class (**B**), family (**C**) and genus (**D**).

A different bacterial distribution was found in each cave (Fig. 3A–D). For instance, the phylum Pseudomonadota dominated throughout the dataset, with remarkable presence of its main classes (Gamma, Alpha, Beta and Delta-Proteobacteria) and by different genera reported in the top 15 (Fig. 3). The class Gamma-Proteobacteria was mainly represented by the genus *Pseudomonas,* particularly abundant in tourist caves (ranging from 11.1% in Bossea to 17.78% in Pertosa-Auletta, vs. Costacalda 2.95%). Conversely, the genera *Sphingomonas* (class Alpha-Proteobacteria, 2.46%), *Lysobacter* (class Gamma-Proteobacteria, 2.34%) and *Polaromonas* (class Beta-Proteobacteria, 1.8%) were prevalent in the pristine site, while *Hyphomicrobium* genus (class Alpha-Proteobacteria) was largely spread across all habitats (average of 0.6%). Three Acidobacteria sub-groups, i.e. Gp6 (mean 6.0%; highest in Bossea: 8.2%), Gp17 (mean 2. 0%), Gp16 (mean 1.74%), Gp4 (mean 1.35%) and the two main Actinobacteriota genera, i.e. *Gaiella* (with 0.78% in Pertosa-Auletta up to 4.51% in Vento caves) and *Arthrobacter* (ranging from 0.24% in Vento up to 2% in Pertosa-Auletta caves) showed a broad distribution across all caves. Yet, among Actinobacteriota, the genus *Bifidobacterium* was recorded only in show-caves (between 0.3% in Caudano and 2.5% in Pertosa-Bossea caves). However, similar abundance trends across all surveyed sites were also reported for the family Planctomycetaceae (ranging from 2.7% in Vento up to 4.27% in Bossea caves) and the genus *Nitrospira* (between 0.7% for Caudano and 1.75% of Costacalda caves). Other remarkable compositional spectra were represented by Bacillota with classes Clostridia (ranging from 0.48% in Caudano up to 1.62% in Bossea, vs. 0.07% in Costacalda) and Bacilli (from 3.15% in Caudano up to 7.66% in Pertosa-Auletta and 1.81% for Costacalda caves) with genus *Lactobacillus* (1.64% mean in show-caves, vs. 0.007% in Costacalda) all typically found in anthropized settings. Conversely, the *Flavobacterium* genus (Phylum Bacteroidota, 2.47% in Costacalda) was more than 8 times higher in the natural one than in show -caves.

### Archaeal community composition

**Figure 4:**
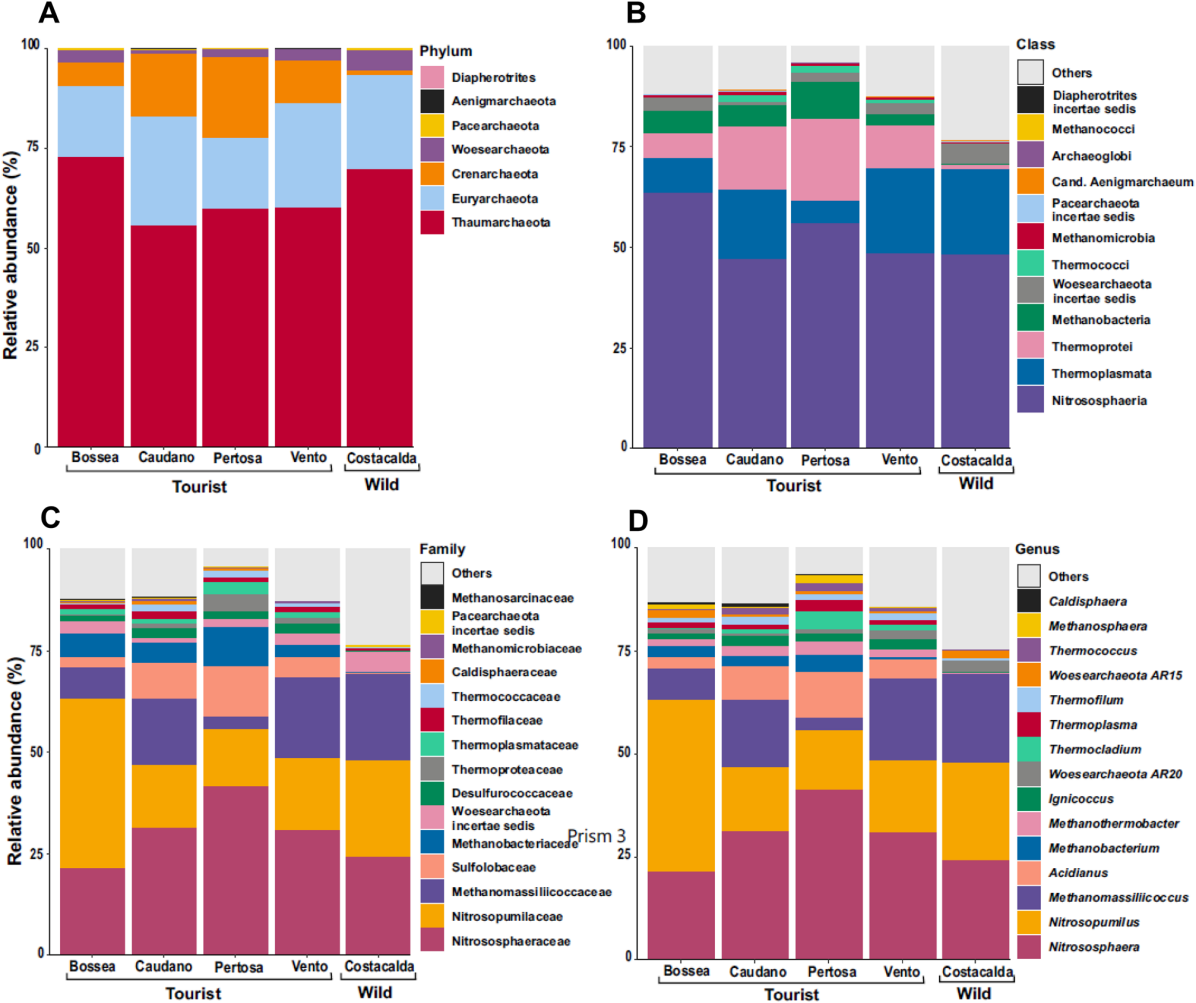
Archaeal community composition of 5 caves studied: Bossea, Caudano Pertosa-Auletta, Vento (show-caves) and Costacalda (wild cave). The top 15 most abundant taxa are shown for taxonomic ranks: phylum (**A**), class (**B**), family (**C**) and genus (**D**).

Archaea showed 2 main compositional trends describing the archaeome throughout the investigated caves (Fig. 4A,B,C,D). The first group is recurrent in all investigated caves and includes Thaumarchaeota and Euryarchaeota as the most abundant phyla, with the genera *Nitrososphaera* (ranging from 21.38% in Bossea to 41.35% in Pertosa-Auletta caves), *Nitrosopumilus* (between 14.23% and 41.76% in Bossea e Pertosa-Auletta) and *Methanomassiliicoccus* (ranging from 3% in Pertosa-Auletta to 21.35% in Costacalda). The same distribution across all caves was observed for taxa in the phylum Woesearchaeota, even if far less represented. The second group included taxa more frequent in show -caves as for the genera *Acidianus* (Phylum Chrenarcheota, class Thermoprotei, ranging from 2.62% Bossea to 11.34% in Pertosa-Auletta), *Ignicoccus* (average in show-caves 2.04%) and *Thermocladium* (4.14% highest value in Pertosa-Auletta); the genera *Methanothermobacter* and *Methanobacterium* (Phylum Euryarcheota, class Methanobacteria) were also found mainly in show -caves, the first with an average of 2.33% and the second with a frequency ranging between 0.55% and 3.9%. Other less abundant genera such as *Methanosphaera* and *Thermococcus* could be included in this second group.

### Micro-algal community composition

**Figure 5:**
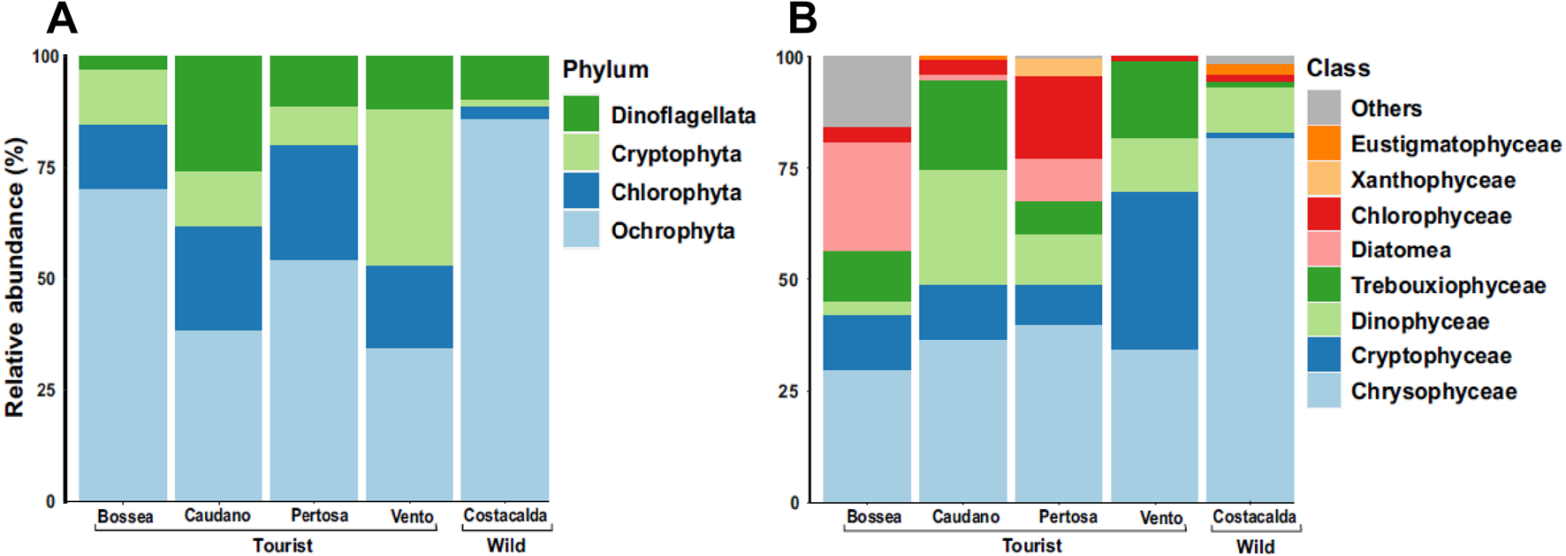
Micro-algal assemblage composition of 5 caves studied: Bossea, Caudano Pertosa-Auletta, del Vento (show-caves) and Costacalda (wild cave). All taxa observed are shown for taxonomic ranks: phylum (**A**) and class (**B**).

Algae is by definition a polyphyletic group; therefore, the micro-algal assemblage (Fig. 5A,B) was derived by clustering members belonging to different kingdoms in order to investigate the micro-photosynthetic component. The compositional scenario was characterized by the Class Chrysophyceae (phylum Ochrophyta) dominating in most of the caves, in particular in Costacalda (81.5%) and Pertosa-Auletta (39.76%) caves. Vento cave was dominated by Cryptophyceae (35.3%) members of the phylum Cryptophyta. Thereafter, community composition became patchy as no other widespread classes were found across the dataset. For instance, Dinophyceae showed a high abundance in the Caudano cave (25.86%), but it dropped in Vento (12.1%), Pertosa-Auletta and Costacalda (11.57%) caves; on the other hand, Trebouxiophyceae showed a decreasing trend along Caudano (20.10%) del Vento (17.37%), Bossea (11.47%) and Pertosa-Auletta (7.25%) caves. The class Diatomea was particularly present in Bossea cave instead (24.48%).

### Shared and unique taxa

**Figure 6:**
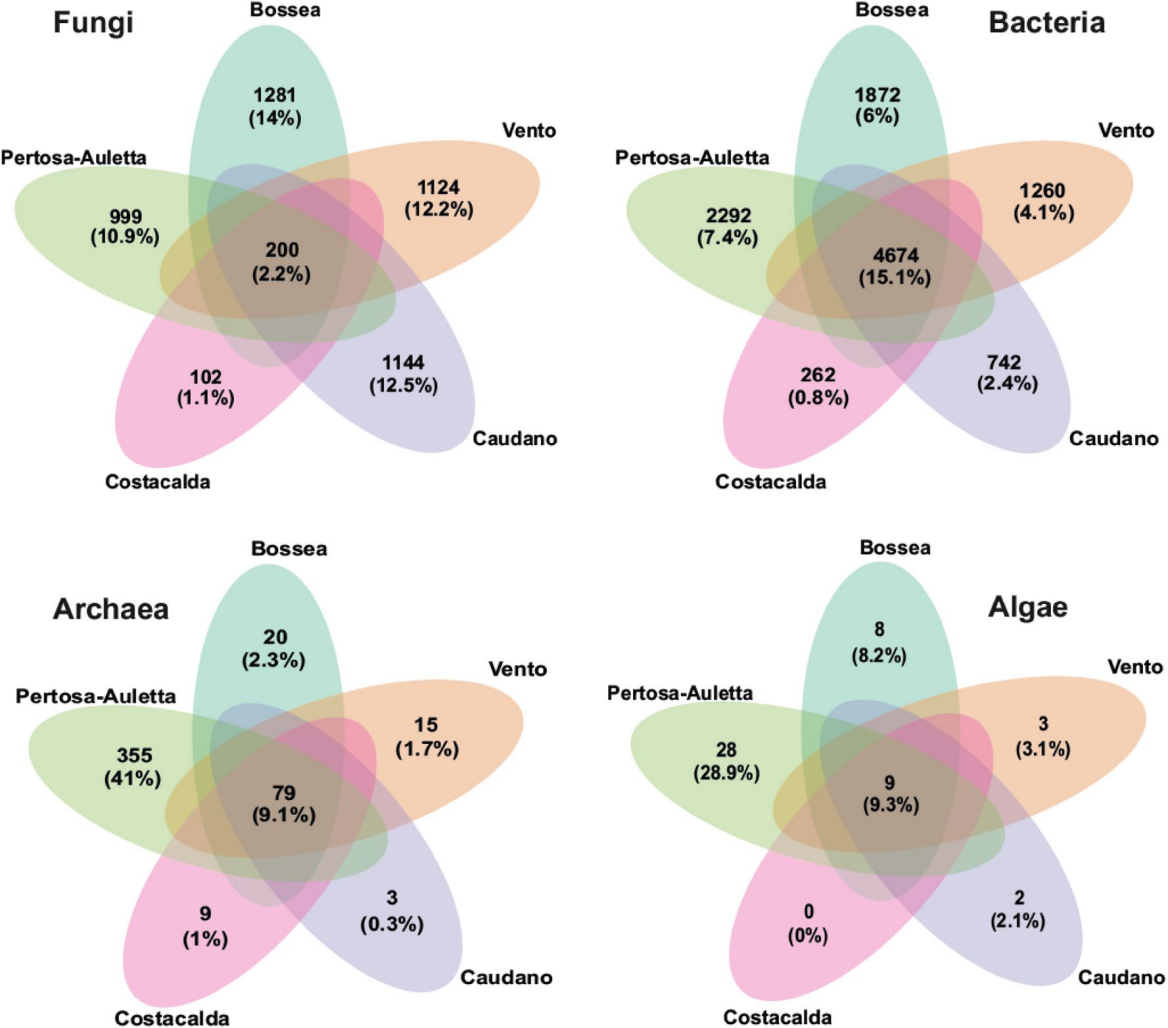
Venn diagrams show shared and unique taxa distribution for each microbial component studied: Fungi, Bacteria, Archaea and Micro-Algae, among the 5 caves investigated: Bossea, Caudano Pertosa-Auletta, Vento (show -caves) and Costacalda (wild cave).

Venn diagrams (Fig. 6) displayed the ASVs that typically characterized each cave (Unique ASVs) and those that are representative of the community core, shared throughout the dataset. Regarding the fungal community, the core was scarcely populated (200 unique ASVs), accounting for only 2.2% of the total composition. The ASVs shared between all caves were represented by 54 genera; in particular *Mortierella* (13%) and *Candida* (10%). ollowed by *Penicillium* (5.4%), *Humicola* (3.8%), *Cephalotrichum* (3.1%), *Geomyces* (2.3%), *Pseudogymnoascus* (1.5%) and *Aspergillus* (1.5%) genera commonly found in cave environments. When considering the unique ASVs, Bossea cave had the highest number of unique ASVs (1281) representing 14% of the total composition, spread over 178 fungal genera. The other caves, showed a conspicuous number of unique ASVs, in particular the Caudano cave (1144 ASVs, 12.5% of the total), displayed the highest number of genera (192) identified among the typical ASVs, followed by Vento cave showed 1,124 unique ASVs (12.2%) and Pertosa-Auletta cave characterized by 999 unique ASVs (10.9%) with 171 identified genera. The wild Costacalda cave counted 102 unique ASVs (1.1%), with only 30 genera among the site-specific ASVs reported.

The bacterial community (Fig. 6) gravitated around a robust core of 4,674 ASVs (15.1%) and 283 genera, among which *Acidobacteria* Gp6 (15.8%), *Gaiella* (6%) and *Nitrospira* (1.9%) were the most abundant. However, the entire cluster of subgroups-*Acidobacteria* accounted for 37% of the shared ASVs. On the other hand, there were few unique ASVs for each cave, with Pertosa-Auletta cave showing 2,292 ASVs (7.4%), followed by Bossea cave (1,870 ASVs, 6%), Vento cave (1,260 ASVs, 4.1%), Caudano cave (742 ASVs, 2.4%) and Costacalda cave (262 ASVs, 0.8%). Moreover, some peculiar genera were uniquely reported for the different caves, such as *Legionella* (3.3%) in Bossea, *Gemmatimonas* (4.5%) in Caudano, *Lactobacillus* (9.5%) *Desulfomicrobium* (3.8%) and *Clostridium *sensu stricto (3.6%) in Pertosa-Auletta.

The distribution of ASVs belonging to the archaeal component among caves (Fig. 6) was sharply defined. Indeed, while the core group (79 ASVs, 9.1%) and unique ASVs of Bossea (20 ASVs, 2.3%), Vento (15 ASVs, 1.7%), Costacalda (9 ASVs, 1%) and Caudano (3 ASVs, 0.3%) caves were represented by a low number of ASVs, the Pertosa-Auletta cave was characterized by 355 unique ASVs (41%), taxonomically labeled by *Acidianus* (33%) and *Thermoplasma* (14%) genera. However, the genera of archaeal methanogens constituted a solid cluster either across the common core (24.3%) and unique ASVs for each cave.

The distribution of taxa detected for the phototrophic assemblage (Fig. 6) was extremely polarized between caves. One pole was represented by Costacalda Cave for which no unique ASVs were recorded. The other one was the cave of Pertosa-Auletta which accounted for 28 unique ASVs (28.9%), mostly belonging to the Chlorophyceae (39%), Chrysophyceae (32%) and Diatomea (18%) classes.

### Human related microbial taxa

Due to the high abundance of some common human-related microbial taxa found in the show -caves investigated (i.e. Malasseziomycetes class and *Candida* genus for fungi, *Lactobacillus* and *Bifidobacterium* genera for bacteria), we decided to explore the occurrence of less abundant guilds too as potential proxies for direct human impact.

**Figure 7:**
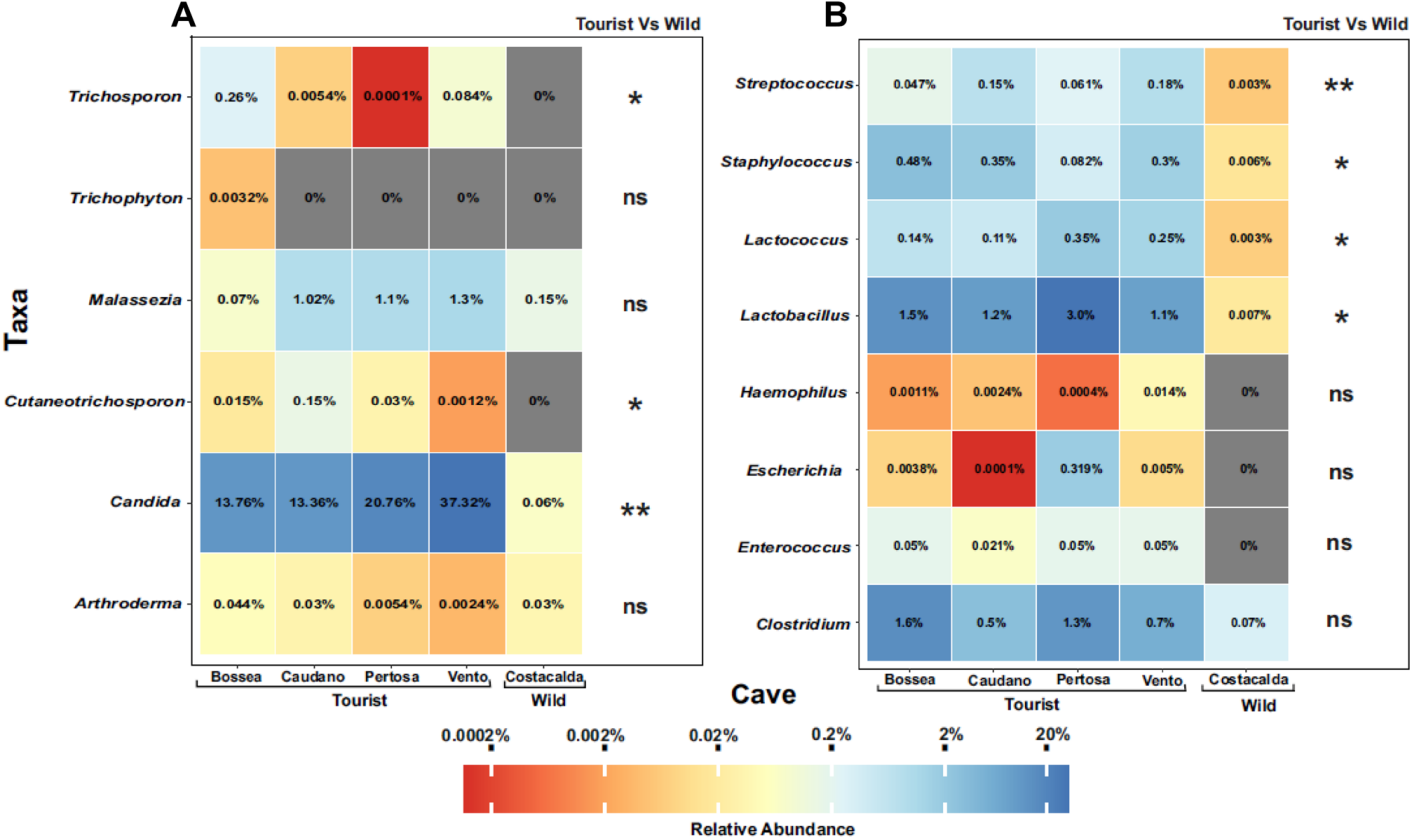
Heatmaps showing the relative abundances of fungal (**A**) and bacterial (**B**) taxa commonly associated to the human body, among the 5 caves investigated: Bossea, Caudano Pertosa-Auletta, Vento (show -caves) and Costacalda (wild cave). Kruskal–Wallis test results comparing tourist caves vs. wild cave are shown as follows: ***(p < 0.001); **(p < 0.01); *(p < 0.05); ns (p > 0.05).

Throughout the fungal dataset (Fig. 7A) tourist caves were especially characterized by an higher significant presence of dermatophytes with the genus *Trichosporon* (Bossea 0.26%, Vento 0.084%, Caudano 0.0054% and Pertosa-Auletta 0.0001%, vs. 0% in Costacalda; p < 0.05) and *Cutaneotrichosporon* on average 0.05% for the show-caves, vs. 0% in Costacalda (p < 0.05). The genus *Arthroderma*, here recorded in all caves analyzed, is instead a geophilic dermatophyte mostly occurring in soil, but also frequent in cave environments, and it is rarely associated with human and animal cutaneous infections^19^. Moreover, *Malassezia* spp., here mainly found in human impacted caves (Bossea 0.07%, Caudano 1.02%, Pertosa-Auletta 1.10% and Vento 1.30%, vs. Costacalda 0.15%; p > 0.05), currently exist as a commensal fungi of the mammalian skin, and associated with atopic dermatitis in inflammatory skin disorders only.

Similarly, for bacterial assemblage (Fig. 7B), many widespread human tissues-inhabiting members of Bacilli class characterized the tourist caves, in contrast to Costacalda wild cave, where their relative abundance never exceeded the 0.007%; in particular *Staphylococcus* (ranging from 0.082% in Pertosa-Auletta to 0.48% in Bossea; p < 0.05), *Streptococcus* (from 0.061% in Pertosa-Auletta to 0.47% in Bossea; p < 0.01), *Lactococcus* (as a mean of 0.21% in the show -caves, vs. 0.003% in Costacalda; p < 0.05) and *Lac tobacillus* (with 1.7% on average in show -caves, vs. 0.007% in Costacalda; p < 0.05) showed a higher significant occurrence within show-caves.

Furthermore, even the *Clostridium* genus (from 0.5% in Caudano to 1.6% in Bossea, vs. 0.07% in Costacalda; p > 0.05) seemed to exhibit a higher occurrence across the anthropized settings.

## Discussion

The effects of alterations occurring on structure, biodiversity, microclimate, energetic condition^11,12^ during the conversion of a natural cave into a show- cave are still almost unexplored. However, the increasing anthropization of new hypogean sites associated with human exploitation constantly impacts cave microbiomes, making their composition and functionalities unpredictable for future conservation and management^9,10,13,20^.

Herein, we deeply investigate, for the first time, microbiomes of four Italian show -caves using next generation sequencing approaches . We provided a first attempt to identify potential human-induced alterations by comparing show -caves with a recently discovered natural cave. The extensive sampling performed, together with the amplicon-sequencing analysis, led to a wide identification of microbial diversity, encompassing 17 phyla, 52 classes and 554 genera for the ITS dataset. On the other hand, the 16S barcode recorded 37 and 7 phyla, 73 and 8 classes, 1,339 and 49 genera for Bacteria and Archaea domains, respectively. For algae , a total of 4 phyla and 9 classes were identified; this low diversity of the phototropic counterpart is likely due to the condition of darkness characterizing the ground sampling area, although abundant algal growths were reported at the level of the rock formations in the examined show -caves^21^.

The mycobiome composition and the distribution of unique fungal ASVs across the 4 surveyed show -caves appeared more complex and diversified than those observed in the natural one. The predominance of *Candida* genus, which includes many species known as common colonizers of humans, in show -caves may reflect a relation with touristic flow. *Candida* has also been reported as an oligotrophic multi-stress tolerant fungus, skilled to colonize a wide range of natural, urbanized and human environmental contexts^22,23^. Cave visitors, as previously reported, may act as primary vector in the spreading of this genus, by bio-aerosols formation (breathing, sneezing or coughing), surface contact and shedding of skin scales, hair and lint from clothing^24^. Potential direct contamination by human-related species^25^ of cave mycobiome are further supported by the higher abundance of dermatophyte fungal genera within tourist caves (e.g. *Cutaneotrichosporon* and *Trichosporon*). Conversely, the mycobiome of the natural cave was dominated by taxa belonging to the genera *Mortierella* and *Cephalotricum*, which include psychrotolerant cellulose-degrading fungi^26,27^, and by the genera *Rodentomyces* and *Gymnoascus*, which include psychrotolerant, coprophilic and keratinolytic species^28,29^, that may be related to the main caves macro-fauna components, i.e. rodents and bats. Furthermore, fast-growing saprophytic fungal genera (*Penicillium*, *Chaetomium* and *Humicola*) were also reported, even if in lower relative abundance.

Such specific fungal traits have been reported as early wild cave colonizers^30^, able to cope with the constraining low-temperature and oligotrophic conditions in these habitats. On the other hand, within the 4 investigated show -caves, the notable abundance of Agaricomycetes (and generally Basidiomycota) may be the result of increasing ventilation during cave opening. In fact, airborne fungal spore contamination is facilitated by the atmospheric continuum between the outdoor and underground environment^31,32^. Moreover, the high occurrence of fast-growing and heavily-sporulating fungi (*Aspergillus* and *Penicillium*), or common competent degraders and plant pathogens (*Chaetomium*, *Phoma*, *Sarocladium* and *Dipodascus*) could be the result of combined effects of tourist flows, spreading fungal alien species and extra complex organic matter input, and the implement of nutrients due to the consumption of food and beverages during events hosted in caves^33,34^. Finally, artificial lighting may have promoted the growth of lichenized fungi (Lecanoromycetes) inside the caves, as reported in previous studies^18,35^. In particular, their colonization of carbonatic rock substrate and speleothems may result in severe aesthetic and structural damage, due to the large extent of thalli and the high lichen acids production. The genus *Tetracladium* was always present in all caves, even if at low frequency. It belongs to the group of “Ingoldian fungi” or aquatic hyphomycetes, phylogenetically diverse fungi growing on decaying leaves and plant litter in streams^36^; their presence in our environmental samples may be related to the presence of water flows present in all caves.

The compositional similarity found throughout the dataset for the Bacteria and Archaea domains, reflects their paramount role in biogeochemical cycles of caves biosphere: C, N, CH_4_-genesis and metabolism and S oxide-reduction, sustaining trophic networks and peculiar caves’ geochemical processes, even in these deeply impacted habitats. In fact, a wide range of polymers degraders particularly adapted to cope with low temperature and oligotrophic conditions were found. For instance, Acidobacteria-subgroups (37% of the shared ASVs among the caves), *Sphingomonas*, *Lysobacter*, *Polaromonas* and a few widespread Mn-Fe oxidizing bacteria, such as family Planctomycetaceae members, *Hyphomicrobium* (Bacteroidota), *Flavobacterium* and *Pseudomonas* (Pseudomonadota) genera. Yet, the *Pseudomonas* high abundance reported in show -caves might be due to the indirect effects of human activities in these habitats. Extra nutrient inputs by tourist flows might stimulate fungal metabolic activity^37^ and lead to high secretion of acids (i.e. oxalate and pyrophosphate), which in turn could trigger the metabolic activity and proliferation of *Pseudomonas* by chelating Mn^38^. Also related to the biogenic carbon cycle, the group of Methane-oxidizing bacteria (MOB) found along α,β,γ-Proetobacteria classes were widely present throughout the caves, underlining their key role in continuous CH_4_ consumption in these subterranean ecosystems^39^. In contrast, *Methanobacterium* and *Methanothermobacter* were more present in tourist caves among the most abundant genera of the Archaea-methanogens counterpart. This compositional alteration could be the result of the indirect effects of tourist flows, which may increase indoor CO_2_ levels up to 200%^40^, promoting the proliferation of these microorganisms, which use H_2_ to reduce carbon dioxide molecules into methane. Compositional variations for the archaeal community have already been reported in touristic caves, due to anthropic pressure and after simulated organic matter treatment on cave microcosms^13,41^. The similar compositional pattern throughout the dataset of the main bacterial and archaeal players involved in the N cycle, i.e. *Nitrospira* (nitrite-oxidant), *Gaiella* (nitrate-reducing), *Nitrososphaera* and *Nitrosopumilus* (ammonia-oxidant), underlines how human cave activities did not impact these microbial traits. Besides, some bacterial taxa could be also considered as bioindicators of human presence being a result of contamination from the entrance^30,42^. For instance, many human-related genera as, *Lactobacillus* (also as unique ASVs), *Lactococcus*, *Legionella* (only as unique ASVs), *Staphylococcus* and *Streptococcus* were mainly, or in some cases uniquely, found in show -caves. Similarly, the recurrence of opportunistic human pathogenic fungi such as *Candida* and dermatophytes (i.e. *Trichosporon* and *Cutaneotrichosporon*) throughout tourist caves, emphasize a probable direct mycobiome cave contamination as the result of the extensive human exploitation of these subterranean settings.

Concerning microalgae, except for a widespread presence among caves of Ochrophyta phylum and gold-brown algae Chrysophyceae related class, the eukaryotic photosynthetic abundance background appeared patchy. Some caves were largely characterized by presence of particular classes, e.g. Bossea cave by Diatomea, Caudano by Dinophyceae and Trebouxiophyceae, Pertosa-Auletta by Chlorophyceae and Vento cave with Cryptophyceae and Trebouxiophyceae. However, the only notable differences were related to the relative abundance values and lack of unique ASVs of microalgal components harbored by the wild Costacalda cave, dominated by the class Chrysophyceae. This class is composed of taxa that prefer oligotrophic conditions and most species in this group show a mixotrophic metabolism, being able to shift between photosynthesis and ingesting smaller organisms or particles for food. We may hypothesize that the micro-algal component in pristine caves was based on a poorly biodiverse common core^18^, populated by species well adapted to cope with the typical challenges of the cave environment, such as strict oligotrophy and darkness^43^. The subsequent anthropization (artificial lighting and tourist flows) of these settings would have had a key role, both in importing allochthonous species and spreading the local and alien microalgal component even in cave’s zones previously uncolonized^18,44^. Yet, the ASVs cores recorded in bacteria, archaea and algae indicate a high degree of adaptation and specialization for these microbial compartments in caves. Conversely, the narrow core observed for the fungal component may indicate a less strict adaptation to the cave environment and broad capacity to colonize different habitats, according to the high physiological, metabolic and stress-tolerance plasticity of these organisms.

In conclusion, this study provides for the first time a multi-spatial and extensive microbiota characterization of 5 Italian caves, among which 4 tourist and 1 pristine cave, shedding light on microbial diversity of different rapidly evolving subterranean environments. We highlighted how the 4 investigated show -caves share common microbial traits, by filtering microorganisms derived from the outside or human-related, multi-stress tolerant or capable of exploiting the extra supplies of organic matter or degradable compounds provided by tourist flows. Although human activities have been affecting these caves for a long time, the principal common microbial traits, related to the biogeochemical key processes, are still clearly detectable. Finally, we have provided some insights into the potential direct microbiome cave contamination by human-related microbial species. It is worth expanding this dataset by sampling additional underground environments, including both show-caves and wild caves, at local (Italian) geographic scales, to confirm the trends of microbial diversity pattern here found in anthropized caves with respect to the wild one. This study represents the groundwork for further microbiome cave analyses to shed light also on the functional guilds of each microbial component and their distribution and variation in both pristine and impacted settings.

## Materials and methods

### Study area

**Figure 8:**
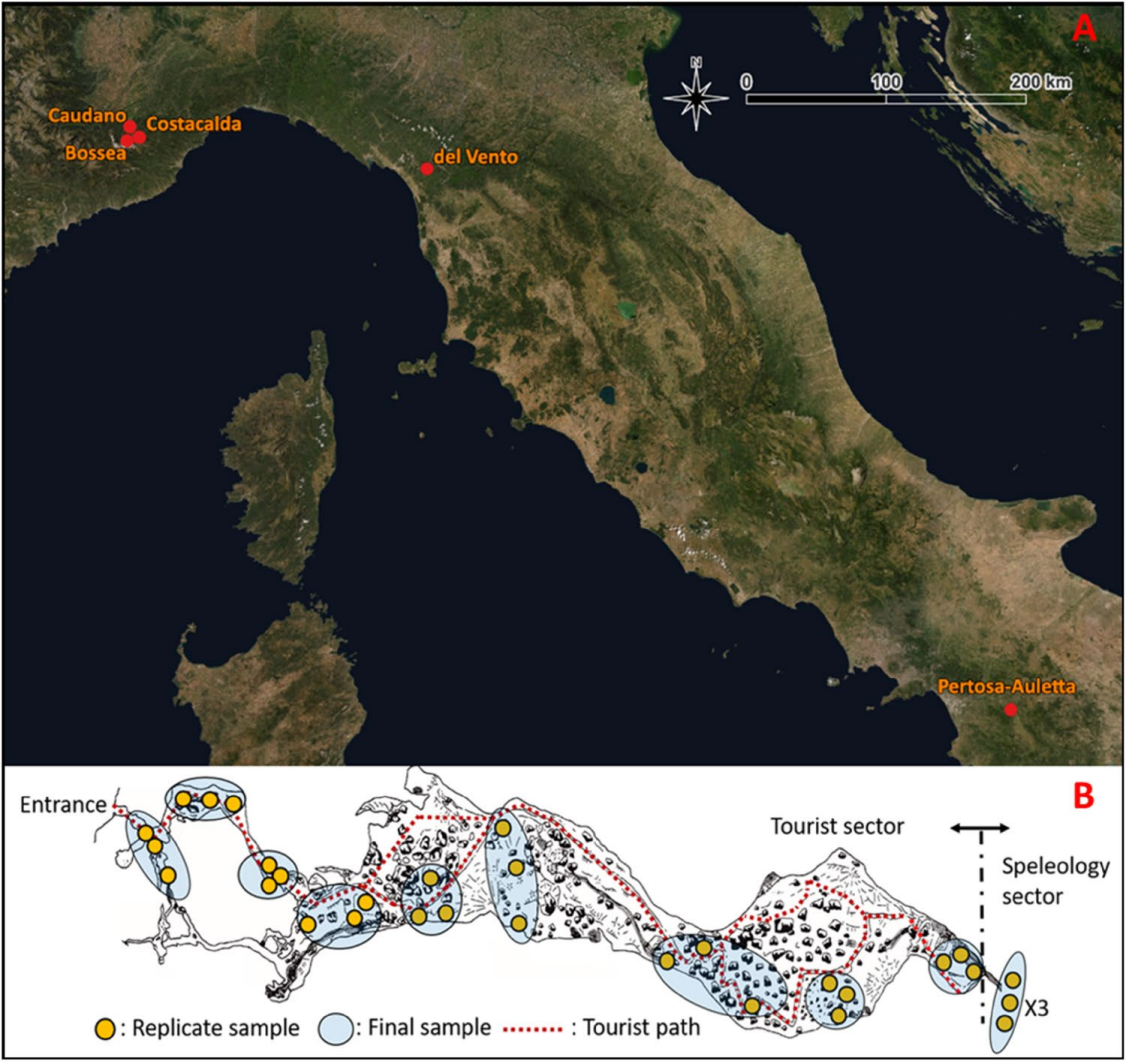
Geographic map of Italy with locations of caves surveyed (**A**) and schematic overview of sampling method applied (**B**). Images were produced mapping the caves’ coordinates layer by qGIS 3.22^45^ on a WMS version of ESRI World Imagery map (https://server.arcgisonline.com/ArcGIS/rest/services/World_Imagery/MapServer/tile/{z}/{y}/{x}).

Five Italian karst caves were selected from north to south Italy (Fig. 8A): the Caudano (44°17′34.3"N; 7°47′25.7"E), Bossea (44°14′31.0"N; 7°50′24.0"E) and Costacalda (44°14′24.8"N; 7°50′54.9"E) caves, in the Maritime Alps complex (Piedmont region); Vento cave (44°02′02.0"N; 10°21′28.2"E) in the Apuan Alps (Tuscany) and the Pertosa-Auletta cave (40°32′14.7"N; 15°27′17.9"E) in the mountain region of the Campanian Apennines.

Besides sharing the same karstic origin, the caves investigated, with the exception of Costacalda, are characterized by a long human exploitation background. Bossea was the first Italian show cave established in 1875 (https://www.grottadibossea.com), while the Caudano cave was permanently opened to the public in 2002 (https://grottedelcaudano.com). Besides regular tourist flows, these caves have experienced exceptional human activity episodes over the last century, like the “700 h underground” experiment (Caudano cave in 1961), where 12 speleologists and some domestic animals lived inside the cave for 1 month, aiming to understand the effect of underground life on human and animals. In addition, Bossea cave has been the setting of many music and food-wine festivals. The Vento cave has been known since the seventeenth century and its cold air currents were used by local people as a natural cooler to preserve food b. The cave was later explored in the nineteenth century and opened to the public for the first time in 1967 (http://grottadelvento.com). The Pertosa-Auletta cave has been exploited by humans since the Neolithic, successively used during the Iron Age (the ruins of a stilt village were found), then converted into a Christian church during the twelfth century and into an air-raid shelter during WWII It was permanently turned into a tourist cave in the second half of the 1900s (https://fondazionemida.com/grotte-pertosa-auletta). Costacalda is a natural (wild; i.e. not impacted) cave discovered in the spring of 2018, when it appeared as a hole about 10 cm wide on the ground. To date, the cave is only partially explored and entirely preserves its pristine structural condition (http://www.speleologiassi.it/82-sommari).

### Sampling design

We applied an extensive sampling of sediments along the whole extension of each cave (Fig. 8B) according to Piano et al. (2022)^20^, starting from the areas near the entrance, towards the deepest zones and at different distances from the tourist/speleological paths. A total of 12 in situ sampling sites were selected for the 4 show -caves, while 3 sites were selected in the Costacalda wild cave. For each sample, 9 technical sediment replicates subdivided into 3 triplets, up to 5 cm depth, were collected using sterile Falcon® tubes (50 ml), for a total of 459 samples. Samples were then stored in a cooler-bag until arrival at the laboratory, where the 3 replicates for each sample were pooled and homogenized. Twelve final samples were assembled for each show cave (Bossea, Caudano, del Vento and Pertosa-Auletta) and 3 for Costacalda, for a total of 51 samples. Sampling collection was carried out between June and September 2020.

### Metagenomic DNA extraction and amplicon sequencing

Sediment samples were sieved, under sterile conditions, by removing coarse rock debris and metagenomic DNA was extracted from 0.5 g of sample using Qiagen DNeasy PowerSoil Pro Kit (Carlsbad, CA, USA). The Internal Transcribed Sequence 1 ribosomal region (ITS1), Eukaryotic SSU rRNA (18S) and hypervariable region V4 of 16S ribosomal gene were targeted to assess the fungal, general eukaryotic and prokaryotic community composition, respectively. The ITS1 region was amplified using barcoded primers ITS1F/ITS2, suitable for shorter read length^46^, the 18S region with the Euk_1391f./EukBr primers (http://www.earthmicrobiome.org), while for the V4 region of 16S, barcoded F515/R806 primer set was used according to Caporaso et al. (2012)^47^. PCRs was performed with 1 μL of each primer, 12.5 μL of Taq DNA Polymerase (Thermo Fisher Scientific Inc., Waltham, MA, USA), 9.5 μL of nuclease-free water (Sigma–Aldrich, St. Louis, MO, USA) and 5 ng of DNA template by an automated thermal cycler (BioRad, Hercules, CA, USA), for a total volume of 25 μL. The ITS1 locus, the eukaryotic SSU rRNA and 16S V4 region were amplified following the protocols and technical specifications according to Coleine et al. (2021)^48^.

Amplicons were quantified by a Qubit dsDNA HS Assay Kit (Life Technologies, Carlsbad, CA, USA) and then pooled. Paired-end sequencing (2 × 300 bp) was carried out on an Illumina MiSeq platform.

### Bioinformatic processing data and downstream analysis

Demultiplexed ITS, 18S and 16S sequence datasets were processed using AMPtk^49^ v.1.5.4 software. Briefly, reads were merged using VSEARCH 2.21.1^50^ and barcodes/indexes and primer sequences were removed from raw data; reads were then subjected to quality trimming to a maximum of 250 bp and discarding reads less than 100 bp in length, and sequencing artifacts were dropped by using USEARCH v.9.1.13 with default parameters^51^. Sequence quality filtering was performed with the expected error parameter of 0.9^52^ and the cleaned reads were clustered at 99% similarity using DADA2 denoising algorithm^53^ that uses a statistical error model to correct sequencing errors to infer the Amplicon Sequence Variants (ASVs). Global singletons and rare taxa (< 5 reads) were skipped as likely false positives due to sequencing errors, following Lindahl et al. (2013)^54^. Finally, taxonomic identification was performed with a sequence identity of 97% as threshold, using hybrid database Global Alignment and SINTAX^51^ on reference databases UNITE 8.2.^55^ and RDP 11^56^.

All samples from each cave were analyzed as replicates to describe the global microbial diversity of each underground environment. The relative abundance data shown in this paper represent the average of the abundance values among all samples for each cave. Kruskal–Wallis test was used to assess significant abundance differences among tourist and natural settings, for the potential human-associated microbial taxa. All analyses were performed using the following R packages: “phyloseq”^57^, “microeco”^58^.

## Supplementary Information

Supplementary Table S1.

Supplementary Table S2.

Supplementary Table S3.

Supplementary Table S4.

## Data Availability

Raw sequences were deposited in Figshare’s repository and are openly available at 10.6084/m9.figshare.21354537.
